# The Development of Novel Reverse Transcription Loop-Mediated Isothermal Amplification Assays for the Detection and Differentiation of Virulent Newcastle Disease Virus

**DOI:** 10.3390/ijms241813847

**Published:** 2023-09-08

**Authors:** Hye-Soon Song, Hyeon-Su Kim, Ji-Ye Kim, Yong-Kuk Kwon, Hye-Ryoung Kim

**Affiliations:** Avian Disease Division, Animal and Plant Quarantine Agency, Gimcheon 39660, Republic of Korea; hssong1217@korea.kr (H.-S.S.); stevenkim0401@gmail.com (H.-S.K.); jiyekim@korea.kr (J.-Y.K.); kwonyk66@korea.kr (Y.-K.K.)

**Keywords:** reverse transcription loop-mediated isothermal amplification, Newcastle disease virus, virulent, differentiation, diagnosis

## Abstract

Newcastle disease (ND) is a highly pathogenic viral infection of poultry with significant economic impacts worldwide. Despite the widespread use of vaccines, ND outbreaks continue to occur even within vaccinated poultry farms. Furthermore, novel Newcastle disease virus (NDV) genotypes are emerging in poultry, increasing the need for the development of rapid, accurate, and simple diagnostic methods. We therefore developed two novel sets of visual reverse transcription loop-mediated isothermal amplification (RT-LAMP) assays based on highly conserved regions of the *HN* and *F* genes. The limits of detection of the NDV-Common-LAMP assay, for all the NDV strains, were 10^3.0^ EID_50_/0.1 mL for Kr005 and 10^2.0^ EID_50_/0.1 mL for Lasota within 35 min. The sensitivity of the NDV-Patho-LAMP assay, used for the strain differentiation of virulent NDV, was 10^2.0^ EID_50_/0.1 mL for Kr005. No amplification was detected for the non-NDV templates. Next, we probed 95 clinical strains and 7 reference strains with the RT-LAMP assays to assess the feasibility of their use in diagnostics. We observed no cross-reactivity across the 102 strains. Furthermore, there was 100% congruence between the RT-LAMP assays and full-length sequencing of the target genes, indicating the potential for visual RT-LAMP in the identification and differentiation of NDV. These novel RT-LAMP assays are ideally suited for the field or resource-limited environments to facilitate the faster detection and differentiation of NDV, which can reduce or avoid further spread.

## 1. Introduction

Newcastle disease virus (NDV), the etiological agent of Newcastle disease (ND), causes a highly pathogenic viral infection in poultry. NDV is an avian type I paramyxovirus (APMV-1) of the genus *Avulavirus*, belonging to the family *Paramyxoviridae* [1,2].

APMV-1 is separated into two distinct clades, class I and class II. Almost all class I viruses are weakly virulent and have been isolated mostly from wild birds (waterfowl and shorebirds) and live bird markets (LBMs) [3]. In contrast, class II viruses are highly virulent and have been found in poultry, pets, and wild birds [4,5].

Class II viruses are further divided into 21 genotypes (genotype I-XXI) and 5 pathotypes (viscerotropic velogenic, neurotropic velogenic, mesogenic, lentogenic, and asymptomatic) based on their clinical signs and pathological lesions [6,7,8]. The virulence of NDV strains is defined using in vivo infections, which measure the intracerebral pathogenicity index (ICPI) in 1-day-old chicks, the mean death time (MDT) in 9-day-old serum pathogen-free (SPF) embryonated chicken eggs, and the intravenous pathogenicity index (IVPI) in 6-week-old SPF chickens [2,9]. These methods are time-consuming, expensive, and laborious and raise ethical considerations for use in routine diagnostics.

The virulence of NDV has been predicted from amino acid sequences at the F protein cleavage site (FCS) using molecular techniques according to the definitions of the World Organization for Animal Health (WOAH) [10]. In most highly virulent velogenic and intermediately virulent mesogenic viruses, the FCS sequence is 112(R/K)-R-Q-(R/K)-R↓F117 (R, arginine; K, lysine; Q, glutamine; F, phenylalanine; arrow, cleavage position; number, residue position in F protein), while the sequence from lentogenic and asymptomatic viruses with low virulence is 112(G/E)-(R/K)-Q-(G/E)-R↓L117 (G, glycine; E, glutamic acid; R, arginine; K, lysine; Q, glutamine; L, leucine; arrow, cleavage position; number, residue position in the F protein).

Most available NDV diagnostic techniques employ variations of reverse-transcription (RT) PCR and real-time quantitative reverse-transcription (qRT) PCR assays, which allow for sensitive detection and pathotype differentiation [11,12,13,14]. Subsequently, PCR products amplified via RT-PCR are analyzed by performing gel electrophoresis, sequencing, a heteroduplex mobility assay, or restriction endonuclease analysis. Additionally, due to the widespread use of NDV vaccine strains and asymptomatic NDV carriage in wild birds, these assays are not suitable for all field samples. Although qRT-PCR allows for fast and high-throughput detection by avoiding a post-PCR processing step, this method strongly relies on trained personnel and expensive laboratory apparatus connected to a stable power supply, limiting its use in many diagnostic laboratories [15,16]. There is therefore a pressing need to develop simple, more rapid, cost-effective, and sensitive detection tools for screening NDV infection.

Loop-mediated isothermal amplification (LAMP) is a nucleotide amplification technique with two or three sets of complementary primers that is conducted within an hour under isothermal conditions [16]. In this study, we successfully developed and evaluated two sets of reverse-transcription loop-mediated isothermal amplification (RT-LAMP) assays, accomplished in one step by adding a reverse transcriptase. The NDV-Common-LAMP assay for NDV and NDV-Patho-LAMP assay for virulent NDV, using highly conserved hemagglutinin-neuraminidase (HN) and fusion (F) NDV sequences, can detect and differentiate NDV for class II.

## 2. Results

### 2.1. Optimization of RT-LAMP Assays

Several LAMP primer sets targeting the *HN* and *F* genes were screened using varying primer concentrations and incubation times to optimize the RT-LAMP conditions. Increasing the concentrations of the FIP and LF primers targeting the *F* gene, we generated RT-LAMP products at low RNA template concentrations (Supplementary Figure S1). Furthermore, to successfully amplify and differentiate the genomic RNA of virulent NDV, incubation at 64 °C for more than 35 min was required. After RT-LAMP amplification under isothermal conditions, a color change from pink to yellow indicated a positive reaction. The sequences of the optimized RT-LAMP primer sets are shown in Table 1.

### 2.2. Specificity of RT-LAMP Assays

**Figure 1:**
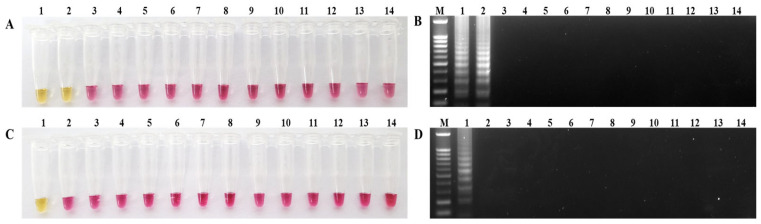
Specificity of NDV-Common-LAMP and NDV-Patho-LAMP assays. (**A**) NDV-Common-LAMP and (**C**) NDV-Patho-LAMP only showed a visual color change from pink to yellow in tubes containing the target genomic RNA. (**B**) NDV-Common-LAMP and (**D**) NDV-Patho-LAMP reactions resolved via 1.5% agarose gel electrophoresis. Lane M, 100 bp DNA size marker; lane (tube) 1, APMV-1(Kr005); lane (tube) 2, APMV-1(Lasota); lane (tube) 3, APMV-2; lane (tube) 4, APMV-3; lane (tube) 5, APMV-4; lane (tube) 6, APMV-6; lane (tube) 7, APMV-8; lane (tube) 8, APMV-9; lane (tube) 9, AIV(H_9_N_2_); lane (tube) 10, AEV; lane (tube) 11, IBDV; lane (tube) 12, IBV; lane (tube) 13, ARV; lane (tube) 14, negative control.

The specificity of the RT-LAMP primers was determined using 50 ng of genomic RNA extracted from APMV-1 (including Kr005 and Lasota), APMV-2, APMV-3, APMV-4, APMV-6, APMV-7, APMV-8, APMV-9, AIV H_9_N_2_, AEV, IBDV, IBV, and ARV. Only tubes containing viral RNA from Kr005 and Lasota and the NDV-Common-LAMP primers displayed a color change from pink to yellow, while the mixtures in the other tubes remained pink (Figure 1A). Additionally, the tube containing RNA from Kr005 and the NDV-Patho-LAMP primers displayed a color change from pink to yellow (Figure 1C). No amplification was observed in the other tubes. Positive reactions were also confirmed via electrophoresis on 1.5% TAE agarose gel (Figure 1B,D).

### 2.3. Sensitivity of RT-LAMP Assays

**Figure 2:**
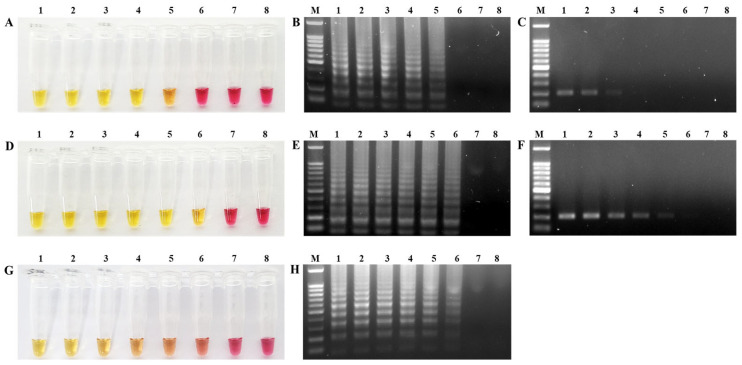
Detection limits of RT-LAMP and RT-PCR assays. (**A**,**D**) Visualization of NDV-Common-LAMP products using Kr005 and Lasota genomic RNA, respectively. (**G**) Visualization of the NDV-Patho-LAMP assay using Kr005 genomic RNA. LAMP-positive reactions display a color change from pink to yellow. (**B**,**E**,**H**) Agarose gel electrophoresis of LAMP products. (**B**) NDV-Common-LAMP for Kr005, (**E**) NDV-Common-LAMP for Lasota, and (**H**) NDV- Patho-LAMP for Kr005. (**C**,**F**) Conventional RT-PCR products resolved via 1.5% agarose gel electrophoresis to compare the sensitivity of RT-PCR with that of the NDV-Common-LAMP assay. Lane M, 100 bp DNA size marker; lane (tube) 1, 10^7.0^ EID_50_/0.1 mL; lane (tube) 2, 10^6.0^ EID_50_/0.1 mL; lane (tube) 3, 10^5.0^ EID_50_/0.1 Ml; lane (tube) 4, 10^4.0^ EID_50_/0.1 mL; lane (tube) 5, 10^3.0^ EID_50_/0.1 mL; lane (tube) 6, 10^2.0^ EID_50_/0.1 mL; lane (tube) 7, 10^1.0^ EID_50_/0.1 mL; lane (tube) 8, negative control.

The limit of detection for the NDV-Common-LAMP and RT-PCR assays was determined via the amplification of serially diluted viral RNA from velogenic (Kr005, from 10^7.0^ to 10^1.0^ EID_50_/0.1 mL) and lentogenic strains (Lasota, from 10^7.0^ to 10^1.0^ EID_50_/0.1 mL). Positive amplification in the NDV-Common-LAMP assay was confirmed by a color change from pink to yellow and the presence of ladder-like DNA bands on the 1.5% TAE agarose gels. In the NDV-Common-LAMP assay, we detected target genes in the concentration range of 10^3.0^ EID_50_/0.1 mL for Kr005 and 10^2.0^ EID_50_/0.1 mL for Lasota under isothermal conditions within 35 min (Figure 2A,B,D,E). In contrast, the sensitivities of the RT-PCR assay using the F3 and B3 primers were 10^5.0^ EID_50_/0.1 mL for Kr005 and 10^3.0^ EID_50_/0.1 mL for Lasota (Figure 2C,F). According to the results, the NDV-Common-LAMP assay is 10~100 times more sensitive than RT-PCR for the detection of NDV. The NDV-Patho-LAMP assay amplified the target gene at 10^2.0^ EID_50_/0.1 mL for Kr005 (Figure 2G,H).

### 2.4. Validation of RT-LAMP Assays with NDV Strains

**Figure 3:**
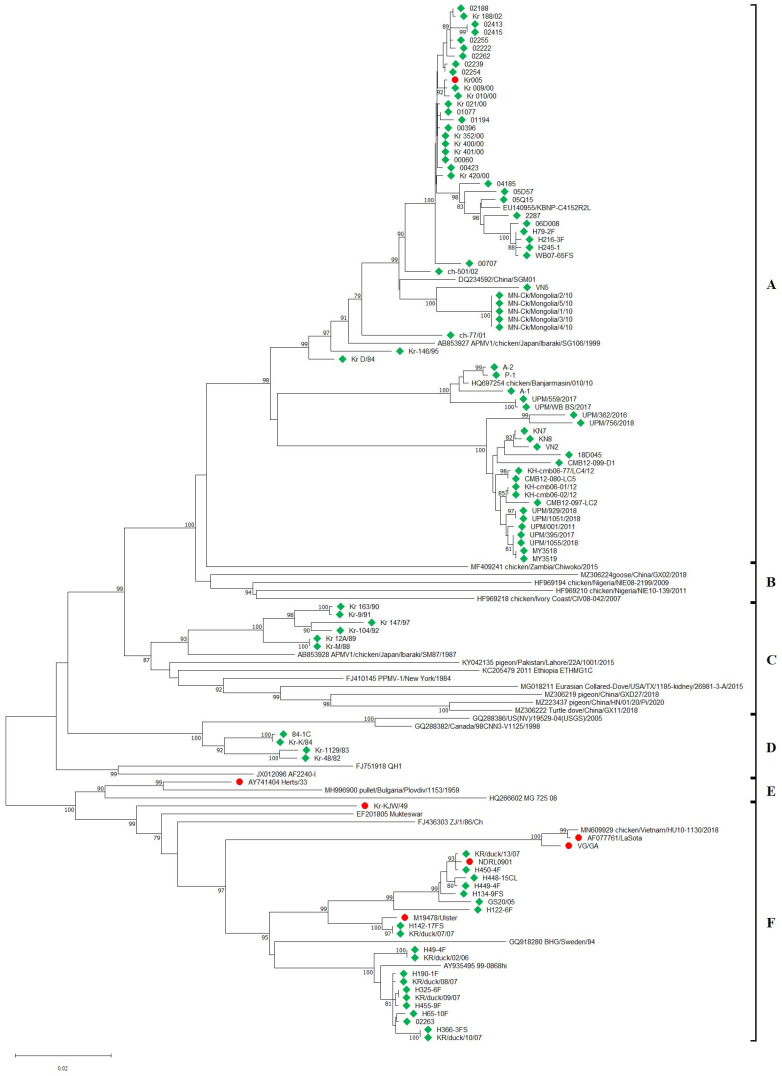
Phylogenetic tree based on full-length sequences of the HN gene constructed using the neighbor-joining method with MEGA (version 11.0). The bootstrap values were determined from 1000 replicates of the original data. Values ≥ 70 are indicated on the branches (as percentages). Six genotypes were identified A, B, C, D, E and F. NDV isolates and reference strains are shown by green squares and red circles, respectively.

**Figure 4:**
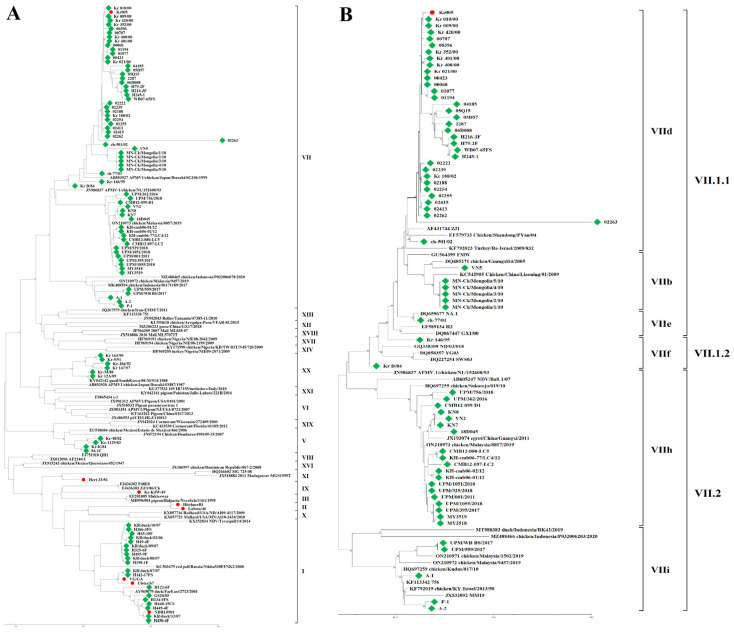
Phylogenetic trees based on the complete F gene using the neighbor-joining method with MEGA (version 11.0). Values ≥ 70 are indicated on the branches (as percentages). (**A**) Phylogenetic analysis of 95 NDV strains (green squares) and seven reference strains (red circles) available in GenBank. (**B**) Phylogenetic tree showing the sub-classification of genotype VII NDV strains (green squares) and a reference strain (red circle).

A total of 102 NDV strains were probed using the NDV-Common-LAMP assay. The amplification results were 100% in agreement with those of the NDV-HN-PCR and NDV-F-PCR assays (Table 2). We subsequently analyzed the amplified PCR products via sequencing. Based on the entire *F* nucleotide sequence of 95 clinical strains, the amino acid sequences (residues 112 to 117) of the FCS were classified into four types: ^112^RRQKRF^117^ for 51 strains isolated from Korea, Vietnam, Pakistan, and Malaysia; ^112^RRRKRF^117^ for 22 strains isolated from Korea, Mongolia, Vietnam, Cambodia, and Malaysia; ^112^KRRKRF^117^ for 2 strains isolated from Malaysia; and ^112^GKQGRL^117^ for 20 strains isolated from Korea. Of the 95 field strains, 75 strains were positive in the NDV-Patho-LAMP assay, and these results were 100% congruent with the amino acid sequences of the FCS. No strain with a ^112^GKQGRL^117^ FCS was detected using the NDV-Patho-LAMP assay. Phylogenetic analysis of the full-length *HN* and *F* genes showed that 95 NDV strains belong to classes A, C, D, E, and F for *HN* (Figure 3) and are grouped into genotypes V, VII, IX, XI, and XX of class II for *F* (Figure 4A). Furthermore, genotype VII was divided into the sub-genotypes VII.1.1 (former VIIb, VIId, and VIIe), VII.1.2 (former VIIf), and VII.2 (former VIIh and VIIi) (Figure 4B). The robustness of branching for the sub-genotypes was supported by high bootstrap values.

**Figure 5:**
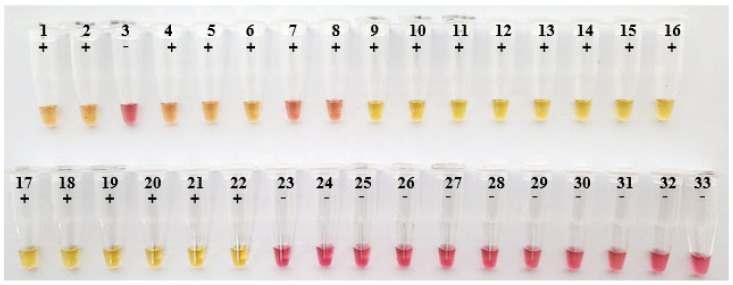
Validation of NDV-Patho-LAMP assay using 32 oropharyngeal swabs. The color of LAMP-positive reactions turned yellow, while the color of LAMP-negative reactions remained pink. Tube 1–8, OP swabs collected from a vaccinated + NDV group; tube 9–22, OP swabs collected from a non-vaccinated + NDV group; tube 23–32, OP swabs collected from a negative group; tube 33, negative control.

As shown in Figure 5, the practical application of the NDV-Patho-LAMP assay using 32 oropharyngeal (OP) swabs showed seven samples as positive among eight samples in the vaccinated + NDV group. All samples in the non-vaccinated + NDV group appeared as positive in yellow, while all samples in the negative control were yielded as negative in pink.

## 3. Discussion

ND was first reported in 1926 in Indonesia and has since spread across the globe and is considered the most dangerous disease of poultry [1]. The WOAH classifies NDV as a list ‘A’ disease, and an outbreak of velogenic or mesogenic ND must be immediately reported to the WOAH, which may result in severe trade restrictions and a major economic burden for the global poultry industry [10]. Although intensive vaccination programs and biosecurity practices have been implemented for decades to limit spread of ND, viral outbreaks, especially those caused by sub-genotype VII.2 (VIIh and VIIi) strains, still sporadically occur in the Middle East, America, Europe, and Africa, in addition to Asian countries including China, Mongolia, Vietnam, Malaysia, and Indonesia [1,5,8,17,18,19,20].

In Korea, the last reported case of ND was in 2010 and was caused by virulent strains of the sub-genotype VII.1.1 (VIId), but no further reports of ND outbreaks have emerged since then. The APQA, which was designated as a WOAH reference laboratory for ND in 2010, has regularly performed active and passive surveillance to monitor the presence or absence of NDV in wild birds, LBMs, and poultry farms, which may be important reservoirs for APMV-1. Here, we established two colorimetric RT-LAMP assays to accurately and rapidly detect NDV and vaccine pathotypes for large-scale NDV screening as well as field testing.

RT-LAMP assays are considered an attractive alternative to PCR-based methods due to their speed, simplicity, specificity, and sensitivity. As RT-LAMP reactions are carried out at a constant temperature of 60–65 °C within an hour, a simple instrument such as a water bath or heat block is sufficient for genomic RNA amplification [21]. Positive results are visually detected as a color change, with no need for agarose gel electrophoresis [22]. Each RT-LAMP assay was optimized under isothermal conditions (64 °C for 35 min) with two sets of primers (six primers each) targeting the *HN* and *F* genes. The *HN* and *F* genes encode surface glycoproteins of the viral envelope, which are responsible for attachment to cell surface receptors and fusion between the cellular and viral membranes [17,23]. Furthermore, since the amino acid sequence of the FCS has a significant impact on fusogenic activity and proteolytic cleavage, the FCS is a key determinant of viral fusion [1].

Our analysis of the specificities of the NDV-Common-LAMP and NDV-Patho-LAMP assays revealed that the target genes were successfully amplified without cross-reactivity with other avian viral pathogens. The limits of detection for the NDV-Common-LAMP and RT-PCR assays using the F3 and B3 primers were estimated using viral genomic RNA from Kr005 and Lasota. This revealed that the sensitivity of the NDV-Common-LAMP assay was much higher than that of a conventional RT-PCR reaction. Furthermore, the NDV-Common-LAMP assay for Lasota was 100 times more sensitive than conventional RT-PCR [24].

In their previous studies, Pham et al. established a LAMP assay for targeting the F gene in 2005, but this method requires a further 2 h for cDNA synthesis and gene amplification [25]. Li et al. developed a one-step reverse transcription (RT)-LAMP assay, in which reverse transcription and amplification were carried out in a single tube [26]. Kirunda et al. developed a convenient and cheaper alternative of RT-LAMP for NDV detection using tracheal tissues and cloacal and oropharyngeal swab samples [27]. In this study, both the specificity and sensitivity of the NDV-Common-LAMP and NDV-Patho-LAMP assays were superior to those reported previously [26,27]. No color changes were observed for the targeting of Hert 33/1956, Kr-KJW/49, and UPM111 using the previous RT-LAMP primers. We compared the sensitivities of the RT-LAMP assays for detecting NDV using 10-fold serial dilutions of RNA templates extracted from Kr005. The detection limits of the RT-LAMP assays developed by Li et al. and Kirunda et al. [26,27]. were the concentrations of 10^−4^ and 10^0^ under isothermal conditions, respectively. These observations indicated that, according to the detection limits of the NDV-Patho-LAMP, it is 10~10,000 times more sensitive than the previous RT-LAMP primers. Furthermore, these previously developed RT-LAMP assays could not distinguish virulent NDV, further highlighting the utility of our assays.

To validate the NDV-Common-LAMP and NDV-Patho-LAMP assays, we performed the amplification of 102 NDV strains and compared the results with those of the Sanger sequencing analysis of *HN* and *F*. We observed 100% (102/102) positivity in the NDV-Common-LAMP, NDV-HN-PCR, and NDV-F-PCR assays when probing the clinical strains. Among the 95 wild strains, the ratio of virulent NDV positivity to the total samples was 78.9% (75/95) using the NDV-Patho-LAMP assay, which was consistent with the FCS amino acid sequences determined from the NDV-F-PCR amplicons. No false-positive or false-negative results were obtained in the 102 strains using either the NDV-Common-LAMP or NDV-Patho-LAMP assay. Furthermore, we also demonstrated that the NDV-Patho-LAMP assay, using OP swab samples, is suitable for screening field samples.

In conclusion, the newly developed NDV-Common-LAMP and NDV-Patho-LAMP assays targeting the *HN* and *F* genes reported in this study are highly sensitive, specific, convenient, and rapid for the detection and differentiation of NDV. Since LAMP reactions can be performed using a battery-driven portable instrument, these RT-LAMP assays have potential utility for on-site diagnosis, even in insufficiently equipped facilities. Our study may assist in the development of prevention and control strategies for NDV in the poultry industry.

## 4. Materials and Methods

### 4.1. Primer Design

NDV-Common-LAMP primers for the simultaneous detection of velogenic, mesogenic, lentogenic, and asymptomatic NDV were designed using Clustal W multiple-sequence alignments of the published target genes in GenBank (http://www.ncbi.nlm.nih.gov (accessed on 1 January 2023)). NDV-Patho-primers specific to virulent NDV were designed to target the variable region. Following the analysis of 100 NDV sequences in the NCBI, we selected the *HN* and *F* genes as regions for the detection of NDV and differentiation of virulent NDV, respectively. The LAMP primers included the following: a forward outer primer F3, a reverse outer primer B3, a forward inner primer FIP (harboring the F2 region at its 3′-end and the F1c region at its 5′-end), a reverse inner primer BIP (harboring the B2 region at its 3′-end and the B1c region at its 5′-end), a forward loop primer LF, and a reverse loop primer LB. The primers recognized eight conserved regions within their target genes and generated amplification products with sizes of 188 bp for NDV-Common-LAMP and 223 bp for NDV-Patho-LAMP, respectively. To validate the LAMP primers for *HN* and *F*, conventional NDV-HN-PCR and NDV-F-PCR primers were designed to amplify full-length sequences of *HN* and *F* (Table 1).

### 4.2. Viral RNA Preparation

Total RNA was extracted from the viral strains APMV-1 (including velogenic Kr005 and lentogenic Lasota), APMV-2 (chicken, Yucaipa, California, 56), APMV-3 (parakeet, Netherlands, 449/75), APMV-4 (duck, Hong Kong, D3/75), APMV-6 (duck, Hong Kong, 18/199/77), APMV-7 (dove, Tennessee, 4/75), APMV-8 (goose, Delaware, 1053/76), APMV-9 (duck, New York, 22/78), avian influenza A (H_9_N_2_) virus (AIV H_9_N_2_, 16AQ075), avian encephalomyelitis virus (AEV, 14ABQ615), infectious bursal disease virus (IBDV, ATCC VR478), infectious bronchitis virus (IBV, ATCC VR21), and avian reovirus (ARV, 15ABD080) using a QIAamp Viral RNA mini kit (Qiagen, Düsseldorf, Germany), according to the manufacturer’s instructions. Viral RNA was eluted in 50 μL of elution buffer and stored at −70 °C until use.

### 4.3. Optimization of RT-LAMP Assays

NDV-Common-LAMP and NDV-Patho-LAMP reactions were carried out in a total volume of 25 μL using a WarmStart Colorimetric LAMP 2× Master Mix (NEB, Ipswich, MA, USA). The reaction mixture contained 2 μL of viral RNA, 2× reaction buffer, 2.5 μM of F3 and B3 primers, 22.5 μM of FIP and 20 μM BIP primers, and 12.5 μM of LF and 10 μM LB primers. The reactions were performed under isothermal conditions at 64 °C for 35 min in a heat block.

### 4.4. Specificity and Detection Limits of RT-LAMP Assays

The specificities of the optimized NDV-Common-LAMP and NDV-Patho-LAMP assays were determined using 50 ng of RNA from APMV-1 (including Kr005 and Lasota), APMV -2, APMV-3, APMV-4, APMV -6, APMV -7, APMV -8, APMV -9, AIV H9N2, AEV, IBDV, IBV, and ARV. Each LAMP reaction was performed at 64 °C for 35 min. The limits of detection of the assays were determined by probing 10-fold serial dilutions of viral RNA extracted from 107.0 EID50/0.1 mL KR005 and 10^7.0^ EID_50_/0.1 mL Lasota. The reaction mixtures were incubated at 64 °C for 35 min. To compare the detection limits of the NDV-Common-LAMP and RT-PCR assays, RT-PCR was performed using F3 and B3 primers (Table 1). PCR amplification was carried out in a 20 μL reaction containing 2 μL of each RNA extract, 2.5 μM of each primer (F3 and B3 of LAMP primers), and 2× reaction buffer (BlackPCR RT-PCR premix; Ventech Science, Daegu, Republic of Korea). The RT-PCR step was 30 min at 45 °C, followed by 5 min at 94 °C. The cycling conditions were 30 cycles of 30 s at 94 °C, 45 s at 55 °C and 72 °C for 30 s, with a final extension at 72 °C for 5 min. The RT-LAMP and RT-PCR products were resolved on 1.5% agarose gel. All dilutions were analyzed in triplicate for the RT-LAMP and PCR assays.

### 4.5. Validation of RT-LAMP Assays with Wild Strains

In total, 95 wild strains (63 from Korea, 2 from China, 6 from Cambodia, 11 from Malaysia, 5 from Mongolia, 5 from Vietnam, and 3 from Pakistan) and 7 reference strains were collected from the Animal and Plant Quarantine Agency (APQA) in Korea from 1946 to 2018. Of the 95 wild strains, 63 strains were isolated from chicken, duck, quail, peafowl, or ostrich in Korea from 1982 to 2008. The other NDV isolates were obtained from chickens, chicken meat, or black swans in neighboring countries. The reference strains consisted of three velogenic viruses (Hert 33/1956, Kr-KJW/49 and Kr005), two lentogenic viruses (Lasota46 and Hitchner B1), and two asymptomatic viruses (Ulster67 and VG/GA). All wild strains were isolated by inoculating the allantois of 9-day-old specific pathogen-free embryonated chicken eggs [10].

The allantoic fluids with hemagglutination (HA) activity (ranging from 2^7^ to 2^10^) were processed for the extraction of viral RNA using a QIAamp Viral RNA mini kit (Qiagen, Germany). These samples were subsequently subjected to NDV-Common-LAMP, NDV-Patho-LAMP, NDV-HN-PCR, and NDV-F-PCR. To analyze the entire *HN* and *F* genes, NDV-HN-PCR and NDV-F-PCR assays were performed in a 20 μL volume containing 2 μL of each RNA extract, 2.5 μM of each primer, and 2× reaction buffer (BlackPCR RT-PCR premix; Ventech Science, Republic of Korea). PCR amplification was performed in a thermal cycler (Eppendorf, Germany) for 1 cycle of 30 min at 45 °C and 5 min at 94 °C, followed by 94 °C for 50 s, 60 °C for 50 s, and 72 °C for 50 s for 37 cycles, before a final extension at 72 °C for 5 min. All amplified fragments were purified using a QIAquick Gel Extraction Kit (Qiagen, Germany), and Sanger sequencing was performed by CosmoGenetech Co. (Seoul, Republic of Korea). The obtained nucleotide sequences were assembled, aligned, and compared using the CLC Main Workbench (Qiagen, Germany). The newly generated datasets were deposited in GenBank (Table 2). Phylogenetic analyses of the complete *HN* and *F* genes were performed using the neighbor-joining method [28] in MEGA 11. The statistical significance of the tree was assessed with a bootstrap value of 1000. In addition, OP swabs were collected from 22 artificially infected chickens at 3 days post-challenge (DPC). Of the 22 chickens, 8 chickens were in the vaccinated + NDV group (highly virulent NDV-challenged chickens after vaccination), and 14 chickens were in the non-vaccinated + NDV group (highly virulent NDV challenged chickens), while 10 chickens were negative controls. The genomic RNA was isolated from OP swabs for the NDV-Patho-LAMP assay.

## 5. Patents

Two patent numbers, 10-2022-0140964 and 10-2022-0140972, for the NDV-Common-LAMP and NDV-Patho-LAMP assays, were approved by the Korean Intellectual Property Office (KIPO) on 28 October 2022.

## Figures and Tables

**Table 1:** Primer sets optimized for the detection and differentiation of Newcastle disease virus.

Primer Set	Target Gene	Primer		Sequence (5′–3′)	Product
					Size (bp)
NDV-Common-LAMP	*HN*	Outer	F3	GGATACCCTCATTCGACATGAG	188
			B3	TTGCACTCACACTGCAAGA	
		Inner	FIP	TCCGAAGCACACAAGTTATACTG	
				CTACATCACAATGTGATA	
			BIP	CATCTCAACAGGGAGGTATCTTCCGATTTTGGGTGTCATC	
		Loop	LF	TGCGAGTGATCTCTGCAACC	
			LB	TCTTTTTACTCTGCGTTCCATCAATCT	
NDV-Patho-LAMP	*F*	Outer	F3	GAGGCATACAATAGAACATGAC	223
			B3	TCTTTAAGCCGGAGGATGTT	
		Inner	FIP	AAGCGTTTTGTCTCCTTCCTCACCC	
				CCCTTGGCGATTCCATCG	
			BIP	TGTAGCTCTTGGGGTTGCAACAGC	
				GCAGCATTCTGGTTGGCTTGTAT	
		Loop	LF	CAGACCCTTGTATCCTGCGGAT	
			LB	GCACAGATAACAGCAGCCGC	
NDV-HN-PCR	*HN*	1643F		ACCCTAGATCAGATGAGAGCC	1643
		1643R		CTACTGTGAGAACTCTGCCTTC	
		1325F		AATCGGAAGTCTTGCAGTGTG	1325
		1325R		TGTGACTCTGGTAGATGATCTG	
NDV-F-PCR	*F*	1333F		GCTAAGTACTCTGAGCCAAAC	1333
		1333R		CAGTATGAGGTGTCGATTCTTCTA	
		1166F		GGGAACAATCAACTCAGCTCATT	1166
		1166R		GCCATGTGTTCTTTGCTTCTC	

**Table 2:** Comparisons of RT-LAMP assays and sequence analysis of *HN* and *F* genes.

Strain		Year of Isolation	Country of Origin	Host	RT-LAMP		Sequencing				
					Common	Patho	Common	Patho		GenBank Accession No.	
							*HN*	*F*	F_0_ Cleavage Site	*HN*	*F*
Reference in NCBI	LaSota/46	1946	-	-	P ^1^	N ^2^	P	P	GRQGRL	AF077761	AF077761
	HitchnerB1	1947	USA	-	P	N	P	P	GRQGRL	-	JN872151
	Ulster/67	-	-	-	P	N	P	P	GRQGRL	AY562991	AY562991
	VG/GA	-	-	-	P	N	P	P	GRQGRL	EU289028	EU289028
	Hert 33/1956	-	-	-	P	P	P	P	RRQRRF	AY741404	AY741404
	Kr-KJW/49	1949	Korea	Chicken	P	P	P	P	RRQKRF	-	AY630409
	Kr005	2000	Korea	Chicken (layer)	P	P	P	P	RRQKRF	-	KY404087
02263		2002	Korea	-	P	N	P	P	GKQGRL	OP921680	OP818810
GS20/05		2005	Korea	-	P	N	P	P	GKQGRL	OP921630	OP818760
KR/duck/02/06		2006	Korea	Duck	P	N	P	P	GKQGRL	OP921712	OP818842
KR/duck/07/07		2006	Korea	Duck	P	N	P	P	GKQGRL	OP921639	OP818769
KR/duck/08/07		2006	Korea	Duck	P	N	P	P	GKQGRL	OP921640	OP818770
KR/duck/09/07		2006	Korea	Duck	P	N	P	P	GKQGRL	OP921641	OP818771
KR/duck/10/07		2006	Korea	Duck	P	N	P	P	GKQGRL	OP921642	OP818772
KR/duck/13/07		2007	Korea	Duck	P	N	P	P	GKQGRL	OP921691	OP818821
H142-17FS		2007	Korea	-	P	N	P	P	GKQGRL	OP921624	OP818754
H190-1F		2007	Korea	-	P	N	P	P	GKQGRL	OP921622	OP818752
H450-4F		2007	Korea	-	P	N	P	P	GKQGRL	OP921625	OP818755
H455-9F		2007	Korea	-	P	N	P	P	GKQGRL	OP921626	OP818756
H448-15CL		2007	Korea	-	P	N	P	P	GKQGRL	OP921627	OP818757
H65-10F		2007	Korea	-	P	N	P	P	GKQGRL	OP921628	OP818758
H449-4F		2007	Korea	-	P	N	P	P	GKQGRL	OP921629	OP818759
H49-4F		2007	Korea	-	P	N	P	P	GKQGRL	OP921634	OP818764
H325-6F		2007	Korea	-	P	N	P	P	GKQGRL	OP921636	OP818766
H122-6F		2007	Korea	-	P	N	P	P	GKQGRL	OP921637	OP818767
H134-9FS		2007	Korea	-	P	N	P	P	GKQGRL	OP921638	OP818768
H366-3FS		2007	Korea	-	P	N	P	P	GKQGRL	OP921632	OP818762
Kr-48/82		1982	Korea	Chicken	P	P	P	P	RRQKRF	OP921713	OP818843
Kr-1129/83		1983	Korea	-	P	P	P	P	RRQKRF	OP921686	OP818816
Kr-M/88		1984	Korea	Quail	P	P	P	P	RRQKRF	OP921718	OP818848
Kr-K/84		1984	Korea	Chicken	P	P	P	P	RRQKRF	OP921687	OP818817
Kr_D/84		1984	Korea	Peafowl	P	P	P	P	RRQKRF	OP921652	OP818782
84-1C		1984	Korea	-	P	P	P	P	RRQKRF	OP921653	OP818783
Kr_12A/89		1989	Korea	Chicken	P	P	P	P	RRRKRF	OP921654	OP818784
Kr_163/90		1990	Korea	-	P	P	P	P	RRRKRF	OP921655	OP818785
Kr-9/91		1991	Korea	-	P	P	P	P	RRRKRF	OP921656	OP818786
Kr-104/92		1992	Korea	-	P	P	P	P	RRRKRF	OP921688	OP818818
Kr-146/95		1995	Korea	Chicken (broiler)	P	P	P	P	RRQKRF	OP921714	OP818844
Kr_147/97		1997	Korea	Chicken (broiler)	P	P	P	P	RRRKRF	OP921657	OP818787
Kr_420/00		2000	Korea	Ostrich	P	P	P	P	RRQKRF	OP921658	OP818788
Kr_009/00		2000	Korea	Chicken (layer)	P	P	P	P	RRQKRF	OP921659	OP818789
Kr_010/00		2000	Korea	Chicken (layer)	P	P	P	P	RRQKRF	OP921660	OP818790
Kr_352/00		2000	Korea	Chicken	P	P	P	P	RRQKRF	OP921661	OP818791
Kr_400/00		2000	Korea	Chicken (broiler)	P	P	P	P	RRQKRF	OP921662	OP818792
Kr_401/00		2000	Korea	Chicken	P	P	P	P	RRQKRF	OP921663	OP818793
00060		2000	Korea	-	P	P	P	P	RRQKRF	OP921664	OP818794
00423		2000	Korea	-	P	P	P	P	RRQKRF	OP921665	OP818795
00707		2000	Korea	-	P	P	P	P	RRQKRF	OP921666	OP818796
Kr_021/00		2000	Korea	Chicken (layer)	P	P	P	P	RRQKRF	OP921667	OP818797
01194		2001	Korea	-	P	P	P	P	RRQKRF	OP921669	OP818799
01077		2001	Korea	-	P	P	P	P	RRQKRF	OP921668	OP818798
02188		2002	Korea	-	P	P	P	P	RRQKRF	OP921670	OP818800
Kr_188/02		2002	Korea	Chicken (broiler)	P	P	P	P	RRQKRF	OP921671	OP818801
00396		2002	Korea	-	P	P	P	P	RRQKRF	OP921672	OP818802
02413		2002	Korea	-	P	P	P	P	RRQKRF	OP921673	OP818803
02415		2002	Korea	-	P	P	P	P	RRQKRF	OP921674	OP818804
02222		2002	Korea	-	P	P	P	P	RRQKRF	OP921675	OP818805
02239		2002	Korea	-	P	P	P	P	RRQKRF	OP921676	OP818806
02254		2002	Korea	-	P	P	P	P	RRQKRF	OP921677	OP818807
02255		2002	Korea	-	P	P	P	P	RRQKRF	OP921678	OP818808
02262		2002	Korea	-	P	P	P	P	RRQKRF	OP921679	OP818809
04185		2004	Korea	-	P	P	P	P	RRQKRF	OP921614	OP818744
05D57		2005	Korea	Chicken (broiler)	P	P	P	P	RRQKRF	OP921715	OP818845
05Q15		2005	Korea	-	P	P	P	P	RRQKRF	OP921620	OP818750
06D008		2006	Korea	Chicken (broiler)	P	P	P	P	RRQKRF	OP921618	OP818748
2287		2006	Korea	-	P	P	P	P	RRQKRF	OP921615	OP818745
H245-1		2007	Korea	-	P	P	P	P	RRQKRF	OP921619	OP818749
H79-2F		2007	Korea	-	P	P	P	P	RRQKRF	OP921623	OP818753
WB07-65FS		2007	Korea	-	P	P	P	P	RRQKRF	OP921631	OP818761
H216-3F		2007	Korea	-	P	P	P	P	RRQKRF	OP921633	OP818763
ch-77/01		2001	China	Chicken meat	P	P	P	P	RRQKRF	OP921651	OP818781
ch-501/02		2002	China	Chicken meat	P	P	P	P	RRQKRF	OP921650	OP818780
MN-Ck/Mongolia/1/10		-	Mongolia	-	P	P	P	P	RRQKRF	OP921699	OP818829
MN-Ck/Mongolia/2/10		-	Mongolia	-	P	P	P	P	RRQKRF	OP921697	OP818827
MN-Ck/Mongolia/3/10		-	Mongolia	-	P	P	P	P	RRQKRF	OP921700	OP818830
MN-Ck/Mongolia/4/10		-	Mongolia	-	P	P	P	P	RRQKRF	OP921701	OP818831
MN-Ck/Mongolia/5/10		-	Mongolia	-	P	P	P	P	RRQKRF	OP921698	OP818828
VN5		2007	Vietnam	Chicken	P	P	P	P	RRQKRF	OP921705	OP818835
VN2		2011	Vietnam	Chicken	P	P	P	P	RRRKRF	OP921716	OP818846
KN7		2012	Vietnam	Chicken	P	P	P	P	RRRKRF	OP921685	OP818815
KN8		2012	Vietnam	Chicken	P	P	P	P	RRRKRF	OP921689	OP818819
18D045		2018	Vietnam	-	P	P	P	P	RRRKRF	OP921690	OP818820
KH-cmb06-01/12		2012	Cambodia	Chicken	P	P	P	P	RRRKRF	OP921643	OP818773
KH-cmb06-02/12		2012	Cambodia	Chicken	P	P	P	P	RRRKRF	OP921644	OP818774
KH-cmb06-77/LC4/12		2012	Cambodia	-	P	P	P	P	RRRKRF	OP921645	OP818775
CMB12-097-LC2		2012	Cambodia	-	P	P	P	P	RRRKRF	OP921647	OP818777
CMB12-080-LC5		2012	Cambodia	-	P	P	P	P	RRRKRF	OP921648	OP818778
CMB12-099-D1		2012	Cambodia	-	P	P	P	P	RRRKRF	OP921649	OP818779
A-1		2016	Pakistan	Chicken (broiler)	P	P	P	P	RRQKRF	OP921702	OP818832
A-2		2016	Pakistan	Chicken (broiler)	P	P	P	P	RRQKRF	OP921703	OP818833
P-1		2016	Pakistan	Chicken (broiler)	P	P	P	P	RRQKRF	OP921704	OP818834
MY3518		2010	Malaysia	-	P	P	P	P	RRRKRF	OP921616	OP818746
MY3519		2010	Malaysia	-	P	P	P	P	RRRKRF	OP921617	OP818747
UPM/001/2011		2011	Malaysia	Chicken (broiler)	P	P	P	P	RRRKRF	OP921681	OP818811
UPM/362/2016		2016	Malaysia	Chicken (broiler)	P	P	P	P	KRRKRF	OP921682	OP818812
UPM/395/2017		2017	Malaysia	Chicken (broiler)	P	P	P	P	RRRKRF	OP921693	OP818823
UPM/559/2017		2017	Malaysia	Chicken (broiler)	P	P	P	P	RRQKRF	OP921683	OP818813
UPM/WB_BS/2017		2017	Malaysia	Black swan	P	P	P	P	RRQKRF	OP921684	OP818814
UPM/756/2018		2018	Malaysia	Chicken (broiler)	P	P	P	P	KRRKRF	OP921692	OP818822
UPM/929/2018		2018	Malaysia	Chicken (broiler)	P	P	P	P	RRRKRF	OP921694	OP818824
UPM/1051/2018		2018	Malaysia	Chicken (layer)	P	P	P	P	RRRKRF	OP921696	OP818826
UPM/1055/2018		2018	Malaysia	Chicken (broiler)	P	P	P	P	RRRKRF	OP921695	OP818825

^1^; positive result, ^2^; negative result.

## Data Availability

The data that support the findings of this study are available from the corresponding author upon reasonable request.

## Supplementary Materials

The supporting information can be downloaded at: https://www.mdpi.com/article/10.3390/ijms241813847/s1.
