# Supplementary material for: The Development of Novel Reverse Transcription Loop-Mediated Isothermal Amplification Assays for the Detection and Differentiation of Virulent Newcastle Disease Virus
Source: Int J Mol Sci. 2023 Sep 8;24(18):13847. doi: 10.3390/ijms241813847 (PMC10531153; doi:10.3390/ijms241813847)
Supplement: Supplementary file 1 [file ijms-24-13847-s001.zip › ijms-2581741-supplementary.pdf]

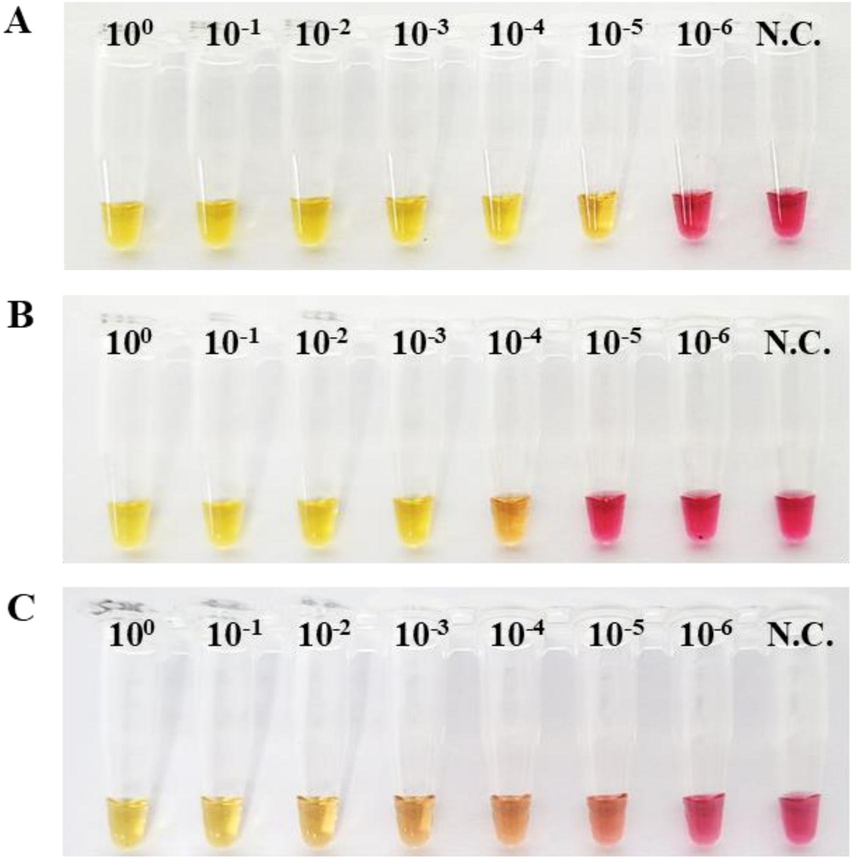

Supplemental Figure S1. Sensitivity of colorimetric NDV-Patho-LAMP assay using 10-fold serial dilutions of genomic RNA at different concentrations of primers [(A) 22.5 μM FIP, 20 μM BIP, 12.5 μM LF, 10 μM LB, 2.5 μM F3, 2.5 μM B3; (B) 22.5 μM FIP, 20 μM BIP, 10.0 μM LF, 10 μM LB, 2.5 μM F3, 2.5 μM B3; (C) 20 μM FIP, 20 μM BIP, 12.5 μM LF, 10 μM LB, 2.5 μM F3, 2.5 μM B3].
